# Is Deprescription of Ezetimibe Safe in Familial Hypercholesterolemia Patients Taking Evolocumab?

**DOI:** 10.1016/j.cjco.2021.12.005

**Published:** 2021-12-17

**Authors:** Shyann Hang, Julieta Lazarte, Robert A. Hegele

**Affiliations:** a Department of Medicine, Schulich School of Medicine and Dentistry, Western University, London, Ontario, Canada; b Department of Biochemistry Schulich School of Medicine and Dentistry, Western University, London, Ontario, Canada; c Robarts Research Institute, Schulich School of Medicine and Dentistry, Western University, London, Ontario, Canada

## Abstract

We evaluated whether low-density lipoprotein cholesterol (LDL-C) levels in familial hypercholesterolemia patients on triple lipid-lowering therapy would remain below intensification threshold values after withdrawal of ezetimibe. We included 13 heterozygous familial hypercholesterolemia patients with vascular disease who were treated with statin + ezetimibe + evolocumab; ezetimibe was discontinued at the patients’ request. After 3 months, LDL-C levels increased from 0.96 ± 0.51 to 1.54 ± 1.07 mmol/L. In 12 of 13 patients, the LDL-C level remained below 1.8 mmol/L. No adverse cardiovascular events were observed. Deprescribing ezetimibe reduced pill burden but increased LDL-C level, although usually not above the treatment intensification threshold for high-risk patients.

## **Résumé**

Nous avons évalué si les concentrations de cholestérol à lipoprotéines de faible densité (C-LDL) des patients atteints d’hypercholestérolémie familiale sur le traitement hypolipidémiant demeureraient en deçà des valeurs seuils d’intensification après le retrait de l’ézétimibe. Nous avons retenu 13 patients atteints d’une maladie vasculaire qui ont la forme hétérozygote d’hypercholestérolémie familiale et qui ont été traités par statine + ézétimibe + évolocumab; l’ézétimibe a été interrompu à la demande des patients. Après 3 mois, les concentrations de C-LDL sont passées de 0,96 ± 0,51 à 1,54 ± 1,07 mmol/l. Chez 12 des 13 patients, les concentrations de C-LDL sont demeurées en deçà de 1,8 mmol/l. Nous n’avons observé aucun événement cardiovasculaire indésirable. La déprescription de l’ézétimibe a permis de réduire le fardeau posologique, mais a entraîné l’augmentation des concentrations de C-LDL, bien qu’elles ne fussent généralement pas au-dessus du seuil d’intensification du traitement des patients exposés à un risque élevé.

Familial hypercholesteremia (FH) is characterized by increased plasma low-density lipoprotein cholesterol (LDL-C) levels, which place untreated patients at high risk of early atherosclerotic cardiovascular disease (ASCVD).^1^ Most cases of FH result from a single pathogenic variant in the gene encoding the LDL receptor (*LDLR*).^1^ The Canadian Cardiovascular Society (CCS) recommends an LDL-C threshold of 1.8 mmol/L for treatment intensification in high-risk patients, such as those with FH.^2^ A similar approach to treatment intensification when LDL-C exceeds 1.8 mmol/L is seen in US lipid guidelines,^3^ whereas European Society of Cardiology (ESC) guidelines recommend a target LDL-C value of 1.4 mmol/L for patients at high risk of ASCVD, including FH patients.^4^

Until recently, strict threshold and target LDL-C values were merely aspirational for FH patients, but this has changed with availability of inhibitors of proprotein convertase subtilisin/kexin type 9 (PCSK9). Statins are first-line therapy in FH and can reduce LDL-C by up to 50%.^2^ If the threshold LDL-C value of 1.8 mmol/L in high-risk patients is exceeded, combination therapy is required, and ezetimibe is usually added.^1^ PCSK9 inhibitors are also very helpful in controlling LDL-C in FH patients, as they incrementally lower LDL-C levels by 50%-60% when added to existing therapies.^1^^,^^2^ Often, all 3 medications are needed to help high-risk FH patients achieve desirable LDL-C thresholds based on treatment.^5^

In our London Health Sciences Centre Lipid Genetics Clinic, FH patients taking maximally tolerated statin plus ezetimibe therapy who then receive a PCSK9 inhibitor often inquire about reducing their oral therapies. We thus evaluated whether deprescribing ezetimibe would maintain LDL-C levels at or below the recommended CCS threshold for intensification of 1.8 mmol/L for high-risk patients. Our secondary objective was to assess whether LDL-C levels remained at or below the ESC target LDL-C level of 1.4 mmol/L.

## Materials and Methods

### Study participants and lipid values

A total of 13 FH patients (9 men and 4 women, mean age 55.4 years) with established ASCVD from our Lipid Genetics Clinic, London Health Sciences Centre (London, Ontario, Canada) were assessed. All data were collected from patient charts. The study was approved by Western University’s ethics board, REB #379.

All 13 patients were taking a maximally tolerated dose of statin, in combination with ezetimibe 10 mg once daily. A total of 12 of 13 patients were on high-intensity statin therapy—8 of 13 were on 40 mg rosuvastatin once daily, 2 of 13 patients were on 40 mg atorvastatin once daily, 2 of 13 were on 20 mg rosuvastatin once daily, and 1 of 13 was on 10 mg rosuvastatin once weekly. For all patients, evolocumab was started at 140 mg subcutaneously biweekly to further reduce LDL-C levels. All 13 patients had requested to stop taking an oral medication after having been on evolocumab. After each patient attained stable LDL-C levels, ezetimibe was deprescribed. Stable LDL-C levels were defined per the clinical judgment of the principal investigator, based on 2 consecutive measurements of LDL-C levels within 10% of each other. Patients were followed at 3 time points, as follows: pre-evolocumab (ie, statin + ezetimibe only); post-evolocumab (i.e., statin + ezetimibe + evolocumab); and after 3 months being off ezetimibe (i.e., statin + evolocumab only). Our outcomes of interest were biochemical changes to total cholesterol (TC), and levels of triglyceride (TG), LDL-C, high-density lipoprotein cholesterol (HDL-C), and non-HDL-C. Pre-evolocumab values were obtained from the most recent results before evolocumab was initiated. Post-evolocumab values were obtained 3 months after evolocumab treatment was started. “Off ezetimibe” values were obtained 3 months after deprescription of ezetimibe.

### Statistical analysis

Statistical analyses were conducted in GraphPad Prism version 9.1.0 (GraphPad Software, San Diego, CA). Percent changes between post-evolocumab and off ezetimibe lipid values were calculated. Comparisons between post-evolocumab and off ezetimibe lipid and lipoprotein values (total cholesterol, non-HDL-C, HDL-C, and LDL-C) were assessed using the Wilcoxon nonparametric test. Triglycerides were assessed using a paired *t* test. Statistical significance was defined as *P* < 0.05. All lipid values are reported as mean ± standard deviation (Table 1).

**Table 1:** Patient demographics and biochemical variables before evolocumab therapy, after evolocumab therapy, and after ezetimibe was deprescribed

Demographics / variables	Pre-evolocumab	Post-evolocumab	Off ezetimibe	*P*	Percent change
Total number / women (%)	13/4 (30.8)	—	—	—	—
Age, y	55.4 ± 11.4	—	—	—	—
Total cholesterol	5.12 ± 0.92	2.86 ± 0.72	3.57 ± 1.22	0.07	24.8
Triglyceride	1.47 ± 0.89	1.51 ± 0.92	1.39 ± 0.72	0.59	–8.0
Non-HDL-C	3.90 ± 1.01	1.62 ± 0.76	2.17 ± 1.31	0.15	34.0
LDL-C	3.24 ± 0.81	0.96 ± 0.51	1.54 ± 1.07	0.06	60.4
HDL-C	1.22 ± 0.35	1.24 ± 0.28	1.40 ± 0.37	0.002	12.9

Percent change refers to “off ezetimibe” compared to “post-evolocumab” values. Values are presented as mean ± standard deviation and are measured in mmol/L, unless otherwise indicated. Post-evolocumab and off ezetimibe values were compared. Pre-evolocumab = statin + ezetimibe; post-evolocumab = statin + ezetimibe + evolocumab; off ezetimibe = statin + evolocumab. HDL-C, high-density lipoprotein cholesterol; LDL-C, low-density lipoprotein cholesterol.

## Results

**Figure 1:**
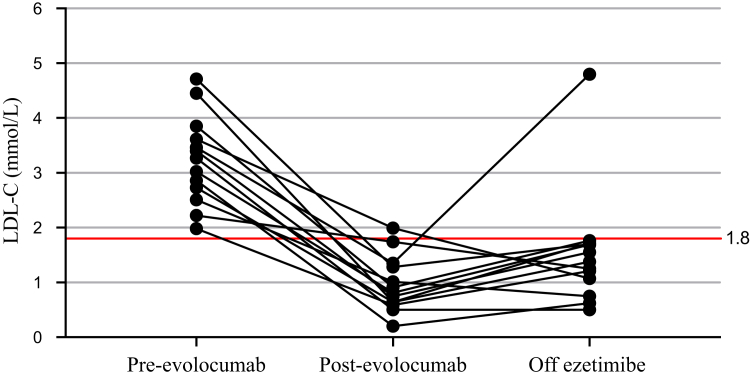
Low-density lipoprotein cholesterol (LDL-C) levels in familial hypercholesterolemia patients at 3 therapy time points: pre-evolocumab = statin + ezetimibe; post-evolocumab = statin + ezetimibe + evolocumab; and off ezetimibe = statin + evolocumab.

Key laboratory variables are shown in Table 1. Overall, 10 of 13 patients had a pathogenic *LDLR* variant—6 were null variants, and 3 were receptor-defective variants. One patient had both a null and defective variant, although phenotypically, he had heterozygous FH. In 3 molecularly uncharacterized patients—2 with no detected variant, and one who had no DNA testing performed—probable FH was diagnosed on clinical grounds. No patient had an elevated polygenic score for LDL-C. Mean ± standard deviation LDL-C levels were 3.24 ± 0.81 mmol/L pre-evolocumab, 0.96 ± 0.51 mmol/L post-evolocumab, and 1.54 ± 1.07 mmol/L off ezetimibe (Table 1; Fig. 1). After withdrawal of ezetimibe, LDL-C level increased in 9 patients, remained stable in 2, and actually decreased in 2 others. HDL-C was increased slightly post-evolocumab and off ezetimibe, whereas other lipid values were not statistically different. Although the percent change in LDL-C level between on and off ezetimibe was 60.4%, when the off ezetimibe LDL-C value was used as the reference point, withdrawing ezetimibe had an ∼35% absolute effect on LDL-C level, which is more in line with the expected effect of ezetimibe. The CCS LDL-C threshold of 1.8 mmol/L was exceeded in one patient (95% confidence interval [CI] of 0 to 5 patients) off ezetimibe. Furthermore, the ESC LDL-C target of 1.4 mmol/L was exceeded in 6 patients (95% CI of 3 to 10 patients) off ezetimibe. No differences according to *LDLR* mutation type were seen. No other distinguishing features were present among the 6 patients with LDL-C > 1.4 mmol/L off ezetimibe, in particular, no apparent differences in age, sex, or baseline lipid profile.

## Discussion

In 13 patients with FH, mean LDL-C levels were not significantly different between patients receiving treatment using statin + ezetimibe + evolocumab and those receiving therapy off ezetimibe—that is, statin + evolocumab only. However, the impact of ezetimibe withdrawal was clinically relevant for individual patients. Specifically, following withdrawal of ezetimibe, one patient (95% CI of 0 to 5 patients) exceeded the CCS recommended LDL-C intensification threshold of 1.8 mmol/L. Furthermore, 6 patients (95% CI of 3 to 10 patients) exceeded the ESC target LDL-C value of 1.4 mmol/L.

Patients whose LDL-C levels exceeded recommended thresholds or targets had no obvious features to account for this. The single patient whose LDL-C level exceeded the CCS intensification threshold had a receptor-defective *LDLR* variant. This patient’s marked increase in LDL-C off ezetimibe could have been due to inconsistent dietary habits, incomplete compliance with medication prescriptions, misunderstanding of medication changes, or anti-drug antibodies that we could not assess. Conditions that raise LDL-C level, such as hypothyroidism, renal disease, and liver disease, were not observed. This patient has since agreed to resume ezetimibe.

Despite our small sample size and observational design, our findings suggest that in a clinically relevant proportion of FH patients, LDL-C might exceed threshold or target values once ezetimibe is withdrawn. A repeat of this study is desirable, with larger patient numbers evaluated over a longer time period, perhaps within the context of a randomized design.

In summary, our findings indicate that if a patient with FH requests deprescription of oral medications subsequent to initiation of PCSK9i therapy, withdrawal of ezetimibe is generally safe. However, if the decision is made to proceed with deprescription, LDL-C levels should still be monitored, and the possibility of restarting ezetimibe should be discussed with the patient.

**Novel Teaching Points**

• Patients with heterozygous FH and an elevated LDL-C level usually require combination therapy.
• Patients on injectable PCSK9 inhibitors often inquire about reducing their use of oral agents.
• After withdrawing ezetimibe in evolocumab-treated patients with heterozygous FH, LDL-C levels rose after 3 months, but they usually did not exceed treatment-intensification threshold values.
